# A Call to Reconsider a Nationwide Electronic Health Record System: Correcting the Failures of the National Program for IT

**DOI:** 10.2196/53112

**Published:** 2023-12-28

**Authors:** James Seymour Morris

**Affiliations:** 1 School of Clinical Medicine, University of Cambridge, Addenbrooke’s Hospital NHS Foundation Trust, Cambridge, United Kingdom

**Keywords:** electronic health record, EHR, medical record linkage, health information interoperability, health information management, health information systems, information systems, interoperability, health records, medical records, national

## Abstract

The National Programme for IT (NPfIT) was launched in 2005 to implement 7 nationwide IT services across the National Health Service (NHS). Despite the success of many of these designated “deliverables,” the establishment of a single nationwide electronic health record (EHR) system never fully materialized. As a result, NHS medical records are now stored using a diverse array of alternate EHR systems, which frequently restricts health care practitioners from accessing extensive portions of their patients’ notes. This not only limits their ability to make well-informed clinical decisions but also impacts the quality of care they are able to provide. This article assesses the medical, economic, and bureaucratic implications of an NHS-wide EHR system. Additionally, it explores how the shortcomings of the NPfIT should be addressed when attempting to introduce such a system in the future.

## “The Biggest IT Failure Ever Seen” [1]

In April 2005, the NHS Connecting for Health Agency (CFH) was established to implement the National Programme for IT (NPfIT). Its goal was to propel NHS England into the 21st century by creating 7 nationwide IT services, including a secure NHSmail system and an electronic prescription service. Although many of these services have now been incorporated into everyday practice, the program’s primary objective was never realized: to establish a single nationwide database of electronic health records (EHRs). Reluctance from several trusts to transition from paper-based records, along with concerns regarding delayed financial returns and data privacy, led to the NPfIT being branded as “the biggest IT failure ever seen” [1], and it was ultimately discontinued. Now, on the decennary of the program’s dissolution, it is increasingly apparent that a nationwide EHR service could represent the panacea for the inexcusable inefficiencies within today’s NHS.

## The Status Quo

Instead of a single nationwide EHR system, NHS trusts currently have the option to choose from 40 different approved suppliers [2]. Due to economic competition, these suppliers offer limited cross-compatibility. NHS staff are well aware of the restrictions this places on the quality of health care. Oftentimes, we cannot access notes from previous admissions due to their being recorded using an alternate EHR system, nor do we have the luxury of previous laboratory results and radiographs with which to compare recent investigations. During the 12 months following April 2017, 3.9 million patients in NHS England attended more than one trust as either an inpatient or outpatient, or in accident and emergency department [3]. On over 11 million occasions, patients sought care at a trust that used a different EHR system than their previous attendance, implying that for 9.1% of all acute presentations [3], clinicians were left uninformed about their patient’s recent medical history.

Following a hospital admission, general practitioners (GPs) are left with little more than a brief discharge summary from which they assimilate weeks’ worth of inpatient notes. To access the results of inpatient investigations, they must first overcome administrative hurdles, and even then, detailed notes are seldom made available to GPs. Although notes can be transferred, most often via GP2GP [4] when switching primary care providers, data are notoriously “lost in translation” between different EHR systems. Furthermore, administrative delays completely preclude the transfer of EHR data in acute health care settings. Such obstacles quickly leave NHS staff feeling not unlike the protagonist of Kafka’s 1926 novel, “The Castle” [5]: so disillusioned by the excessive complexity of bureaucracy that they settle for the status quo, ceasing to seek the information they presume to be unattainable.

Creating a single national database would not only improve the quality of care for contemporary patients but also benefit generations to come. Clinical research would achieve unprecedented statistical power if physicians were granted access to the full cohort of patients registered with NHS GPs—comprising over 62 million people in England alone [6]. Although national research databases are available through the Data Access Request Service, they provide incomplete information of limited applicability and are are hindered by a thick layer of bureaucratic red tape.

Finally, the burden of manually reporting notifiable diseases and maternal deaths could be entirely avoided by automating the process within a nationwide EHR system, thereby improving both the efficiency and fidelity of MBRRACE-UK (Mothers and Babies: Reducing Risk through Audit and Confidential Enquiries) and UKHSA (United Kingdom Health Security Agency) data.

## Lessons From NPfIT

The status quo of NHS record-keeping is indisputably Kafkaesque, but if we are to successfully establish a nationwide database the second time around, we must first address the NPfIT’s shortcomings. The Committee of Public Accounts published a damning report outlining the reasons for the program’s failure; of these, poor financial returns received the lion’s share of the blame. The £9.8 billion investment had yielded just £3.7 billion out of a forecasted £10.7 billion by March 2012 [7] (£1.00 GBP = $1.27 USD at the time of writing); however, this was largely due to the reluctancy of many NHS trusts to switch to the nationwide system, with only 22 of 220 trusts joining the integrated network. Without full participation, the government not only had a weak position in negotiating licensing costs [7], but it was also unable to reap the aforementioned benefits to efficiency and service quality that come with a fully unified system. Therefore, the reported £3.7 billion is likely to represent but a fraction of the potential savings under the final system. The licensing costs for 5 years were originally quoted at £3.1 million per trust [7] (then, £682 million in total), suggesting that the majority of the costs up until the program’s dissolution were one-time investments in establishing the new EHR system, in addition to the £31.5 million squandered on prematurely terminating existing contracts with Fujitsu [7]. The costs quoted are, therefore, likely to diminish alongside exponentially increasing benefits as the system is implemented throughout the NHS.

There have been concerns that granting a single provider with a monopoly on EHRs in the NHS could result in drastic price hikes and limit service quality, with critics quoting the old adage that “competition breeds innovation.” This is true, but only with regard to innovations that maximize profits. By incentivizing co-operation rather than competition between developers (for example, through establishing open source software development), beneficial features would be accreted rather than credentialed as intellectual property, which Carson aptly criticizes as a “toll on the free transfer of information” [8]. In addition, there is evidence to suggest that countries with stricter market entry regulations, and thus, restricted economic competition, do not suffer from reduced quality in public or private goods and services [9].

To motivate universal participation throughout the NHS, the concerns of its employees must also be addressed; namely, the unintuitive user interface and limited functionality of all EHR systems during the early days of their development. This caused many trusts to stick with paper-based records and, as of May 2022, 27 NHS trusts had still not transitioned to digital records [10]. Although the most popular EHR systems are still considered to have suboptimal usability [11], the principal barrier today will be convincing trusts to switch from one EHR to the nationwide system, rather than phasing out paper-based systems, as was the original issue. Parenthetically, EHR system usability varies significantly between different developers [11], and as such, implementing a single nationwide system should also help to curtail geographical health care inequalities by providing a more uniform service.

The NPfIT was further criticized for failing to provide training for NHS staff on how to use the novel EHR systems, which did not initially allow clinicians to view laboratory results and other investigations within the same interface. The former issue is easily resolved by offering training to staff who are unfamiliar with the new EHR system using a platform like e-Learning for Healthcare. Conversely, the latter emphasizes the need for the nationwide EHR system to be a “one-stop shop” for all patient data. The interface should combine both primary and secondary care notes, along with investigations and imaging within a single interface, a concept which, in primary care settings, has already been demonstrated to improve cost-effectiveness, efficiency, and patient satisfaction [12].

Using a single platform to store the comprehensive medical records of an entire population does, nonetheless, bring with it a risk of massive data breaches. Accordingly, the NPfIT and its predecessors were heavily criticized for failing to establish adequate safeguards against such a scenario in which patient data would be vulnerable to surveillance by governmental and clandestine organisations. The unsettling imagery this provokes—that of an Orwellian panopticon endlessly monitoring the public’s medical records—led Privacy International to present the NPfIT with a Big Brother Award for the “most appalling project” of 2004. Since then, the public has grown far more supportive of a nationwide system, with only 9.6% of participants from a 2013 study [13], and 12% of participants from a 2015 study [14], opposing the nationwide EHR system. This shift in attitudes has coincided with increasing safeguards as well as the introduction of mandatory annual training on data protection for all NHS staff. Nevertheless, a unified system should still demand further safeguards, such as limiting the access of the most sensitive sections of the record, for example, genitourinary medicine, fertility, and psychiatry, to those health care professionals directly involved in a given patient’s care.

## Looking to the Future

Switching to a single nationwide system will, of course, demand a massive investment of time and resources; however, should it be attempted, it has the potential to slash NHS expenditure for decades to come. Policymakers will have to garner the support of most, if not all, 215 NHS trusts, if the system is to prove financially viable and avoid falling victim to the same shortcomings as its predecessor. Once a contract is negotiated, a firm date should be set, by which time all NHS trusts are required to phase out their current EHR system in favor of the nationwide network. Such a date should lie beyond the expiry of all contemporary contracts to circumvent the expenses associated with their premature termination, an oversight which ultimately contributed to the downfall of the NPfIT. Fortuitously, the majority of the concerns raised against the program, both by clinicians and the general public, have since been overcome. Nonetheless, e-learning modules should be introduced during the interregnum to allow health care staff to familiarize themselves with the new platform and to emphasize the importance of high-quality data entry prior to its formal introduction. The EHR system itself will have to be refined at regular intervals if it is to continue meeting the ever-evolving needs of its various stakeholders. It is essential, therefore, that a public health body be established to represent the needs of each NHS trust and to continuously re-evaluate them as part of the NHS commissioning cycle. When these are no longer being met, such an organization should present these shortcomings to developers and advocate for specific enhancements to be made to the software.

By providing NHS physicians and approved researchers with a cradle-to-grave record of all NHS patients, the nationwide EHR system would allow clinical decisions to become far better informed and, as a result, the quality of health care should also drastically improve. With consideration for the myriad benefits outlined hitherto, the Department of Health and Social Care is urged not to abandon the nationwide EHR system as Kafka did “The Castle” [5], but to see it through until clinicians and patients alike may reap these benefits for years to come.

## Abbreviations

EHR: electronic health record
GP: general practitioner
MBRRACE-UK: Mothers and Babies: Reducing Risk through Audit and Confidential Enquiries
NHS: National Health Service
NPfIT: National Programme for IT
UKHSA: United Kingdom Health Security Agency
