# Correction: A Call to Reconsider a Nationwide Electronic Health Record System: Correcting the Failures of the National Program for IT

**DOI:** 10.2196/56050

**Published:** 2024-01-12

**Authors:** James Seymour Morris

**Affiliations:** 1 School of Clinical Medicine, University of Cambridge, Addenbrooke’s Hospital NHS Foundation Trust, Cambridge, United Kingdom

In “A Call to Reconsider a Nationwide Electronic Health Record System: Correcting the Failures of the National Program for IT” (JMIR Med Inform 2023;11:e53112) the author noted one error.

In the section titled “The Status Quo,” the following sentence appears:

Clinical research would achieve unprecedented statistical power if physicians were granted access to the full cohort of patients registered with NHS GPs—comprising over 62 people in England alone.

This has been changed to read as follows:

Clinical research would achieve unprecedented statistical power if physicians were granted access to the full cohort of patients registered with NHS GPs—comprising over 62 million people in England alone.

The correction will appear in the online version of the paper on the JMIR Publications website on January 12, 2024 together with the publication of this correction notice. Because this was made after submission to PubMed, PubMed Central, and other full-text repositories, the corrected article has also been resubmitted to those repositories.

